# Supplementary material for: Effects of Insect Cuticular Compounds on Appressorium Formation and Metabolic Activity in Beauveria bassiana
Source: J Fungi (Basel). 2025 Nov 25;11(12):833. doi: 10.3390/jof11120833 (PMC12734190; doi:10.3390/jof11120833)
Supplement: Supplementary file 1 [file jof-11-00833-s001.zip › jof-3974486-supplementary.pdf]

**Table S1**: Spore germination rate and appressorium formation rate of *Beauveria bassiana* in different concentrations of epidermal compound analogue

| Subtance | Concentration (mg/mL) | Germination rate at 12 h (%) | Germination rate at 24 h (%) | Appressorium differentiation rate at 24 h (%) | Germination rate at 36 h (%) | Appressorium differentiation rate at 36 h (%) | Germination rate at 48 h (%) | Appressorium differentiation rate at 48 h (%) | Germination rate at 72 h (%) | Appressorium differentiation rate at 72 h (%) |
|---|---|---|---|---|---|---|---|---|---|---|
| Carnitine C3:0 | 0.05 | 9.00±1.58 b | 30.60±3.71 c | 13.40±2.70 a | 39.00±2.00 c | 24.40±1.14 a | 56.60±2.79 c | 29.80±1.48 c | 80.40±1.34 ab | 37.00±1.87 cd |
| | 0.1 | 13.60±2.19 a | 40.80±1.92 a | 13.80±2.17 a | 56.80±3.27 a | 27.80±2.28 a | 65.00±2.45 a | 44.80±3.42 a | 82.20±1.30 a | 47.00±1.87 a |
| | 0.25 | 8.80±1.48 b | 34.80±2.77 b | 9.80±3.11 b | 44.60±2.19 b | 25.00±1.22 ab | 59.00±1.58 bc | 35.00±2.55 b | 78.80±1.48 b | 39.20±1.92 bc |
| | 0.5 | 4.00±1.00 c | 25.60±3.43 d | 4.60±1.52 d | 35.20±1.92 d | 25.00±3.81 ab | 52.40±2.07 d | 32.40±1.34 bc | 75.00±2.34 c | 36.40±1.67 cd |
| | 1 | 2.00±1.58 c | 24.20±1.79 d | 5.80±3.03 cd | 34.60±1.82 d | 17.40±2.61 c | 49.20±2.59 e | 32.80±1.92 b | 71.20±1.48 d | 35.20±3.90 d |
| | CK | 3.60±0.89 c | 22.40±1.82 d | 8.40±0.55 bc | 40.20±1.64 c | 24.80±1.48 ab | 60.20±1.79 b | 34.60±0.55 b | 71.20±1.92 d | 40.40±1.14 b |
| DOPC | 0.01 | 3.80±2.49 a | 17.40±1.14 bc | 6.60±0.89 ab | 41.80±3.77 ab | 23.60±3.65 ab | 61.20±6.60 a | 34.00±3.32 b | 76.20±4.60 ab | 42.00±2.45 ab |
| | 0.05 | 1.80±1.92 a | 16.00±3.61 cd | 8.40±2.19 a | 41.20±2.77 b | 22.40±3.29 b | 60.60±6.50 a | 35.40±4.67 ab | 68.80±5.40 c | 39.40±2.79 b |
| | 0.1 | 1.60±0.89 a | 13.60±1.82 d | 5.40±0.89 b | 39.80±4.32 b | 22.00±3.16 b | 61.20±3.27 a | 38.40±1.67 ab | 66.4±4.34 c | 38.40±1.67 b |
| | 0.5 | 1.80±1.64 a | 13.40±2.61 d | 5.60±1.52 b | 40.4±2.41 b | 23.80±1.79 ab | 61.00±3.32 a | 36.40±3.85 ab | 66.80±3.90 c | 37.60±4.88 b |

| | | | | | | | | | | |
|---|---|---|---|---|---|---|---|---|---|---|
| DOPC | 1 | 3.40±1.82 a | 20.20±2.77 ab | 5.20±1.92 b | 46.20±5.22 a | 26.60±3.36 a | 61.00±2.55 a | 38.80±3.11 ab | 78.20±2.39 a | 45.00±3.87 a |
| | CK | 3.60±0.89 a | 22.40±1.82 a | 8.40±0.55 a | 40.20±1.64 b | 24.80±1.48 ab | 60.20±1.79 a | 34.60±0.55 a | 71.20±1.92 bc | 40.40±1.14 b |
| Glycylmethionine | 0.05 | 3.40±1.14 a | 20.00±1.58 c | 7.40±1.82 a | 40.20±1.92 a | 25.00±2.92 a | 60.80±3.11 a | 34.00±2.92 b | 70.80± 1.64a | 43.20±2.68 a |
| | 0.1 | 4.00±1.87 a | 18.60±2.07 c | 8.00±3.39 c | 38.40±1.82 a | 27.60±1.82 a | 59.40±2.41 a | 33.60±2.61 b | 70.00±1.41 a | 41.40±1.95 a |
| | 0.25 | 3.40±0.89 a | 20.80±2.49 bc | 10.20±1.30 bc | 40.80±2.17 a | 28.60±2.41 a | 61.20±3.63 a | 38.60±3.51 a | 71.00±3.67 a | 42.60±2.61 a |
| | 0.5 | 2.80±1.64 a | 24.60±3.78 b | 9.40±2.07 b | 40.00±2.00 a | 26.20±3.70 a | 61.20±3.03 a | 34.40±1.67 b | 73.00±3.08 a | 40.20±2.77 a |
| | 1 | 4.40±1.14 a | 29.00±4.74 a | 7.80±2.77 a | 40.40±4.77 a | 15.40±3.44 b | 58.80±4.32 a | 31.80±3.19 b | 72.00±6.40 a | 35.80±4.82 b |
| | CK | 3.60±0.89 a | 22.40±1.82 bc | 8.40±0.55 a | 40.20±1.64 a | 24.80±1.48 a | 60.20±1.79 a | 34.60±0.55 b | 71.20±1.92 a | 40.40±1.14 a |
| TPP | 0.05 | 8.80±1.30 b | 36.20±1.92 a | 12.60±2.70 a | 49.00±2.24 b | 24.00±1.73 a | 59.40±2.88 b | 34.60±2.61 b | 76.80±1.30 b | 40.00±1.58 b |
| | 0.1 | 10.60±3.21 ab | 31.20±3.70 b | 13.80±4.60 a | 54.80±2.17 a | 25.40±2.07 a | 65.40±2.30 a | 40.20±2.77 a | 81.20±1.92 a | 44.80±3.96 a |
| | 0.25 | 12.40±1.95 a | 38.80±3.56 a | 14.80±1.92 a | 49.80±2.68 b | 25.40±5.73 a | 59.40±2.88 b | 32.40±3.21 bc | 76.80±1.30 b | 40.60±3.13 b |
| | 0.5 | 11.20±2.17 ab | 36.80±6.18 a | 13.00±2.92 a | 51.60±1.14 b | 21.80±3.11 a | 59.00±1.87 b | 29.80±3.42 c | 76.80±1.79 b | 42.80±2.86 ab |
| | 1 | 3.40±0.55 c | 10.40±1.67 d | 1.80±0.45 c | 20.00±2.74 d | 5.60±1.52 b | 47.20±2.59 c | 23.20±3.90 d | 56.60±4.62 d | 34.60±4.04 c |
| | CK | 3.60±0.89 c | 22.40±1.82 c | 8.40±0.55 b | 40.20±1.64 c | 24.80±1.48 a | 60.20±1.79 b | 34.60±0.55 b | 71.20±1.92 c | 40.40±1.14 b |
| Enilconazole | $1\times10^{-5}$ | 1.80±0.84 b | 17.40±2.30 b | 4.20±1.48 b | 44.20±2.34 a | 23.20±2.28 b | 51.60±3.58 b | 31.80±2.77 b | 69.80±3.56 a | 42.60±3.05 a |
| | $1\times10^{-4}$ | ND | 13.80±2.28 c | 2.20±0.84 c | 28.60±5.46 c | 18.80±4.44 c | 45.00±4.00 c | 22.40±1.95 c | 62.20±3.42 b | 38.40±6.91 a |

| | | | | | | | | | | |
|---|---|---|---|---|---|---|---|---|---|---|
| Enilconazole | $1\times10^{-3}$ | ND | 1.60±1.52 d | ND | 6.20±1.48 d | ND | 17.20±2.38 d | 2.00±0.71 d | 31.00±3.67 c | 9.20±2.39 b |
| | $1\times10^{-2}$ | ND | ND | ND | ND | ND | 2.20±1.64 e | ND | 3.40±1.14 d | ND |
| | $1\times10^{-1}$ | ND | ND | ND | ND | ND | ND | ND | ND | ND |
| | CK | 3.60±0.89 a | 22.40±1.82 a | 8.40±0.55 a | 40.20±1.64 b | 24.80±1.48 a | 60.20±1.79 a | 34.60±0.55 a | 71.20±1.92 a | 40.40±1.14 a |
| DSBA | $1\times10^{-4}$ | 2.60±0.55 b | 19.00±1.22 b | 3.00±0.71 b | 34.80±1.48 b | 25.60±2.70 a | 55.60±2.70 b | 32.201.30 c | 75.00±2.83 a | 38.00±3.39 b |
| | $1\times10^{-3}$ | 5.80±2.49 a | 22.20±2.28 a | 0.60±0.89 c | 41.20±2.77 a | 17.20±3.70 b | 52.80±2.59 b | 41.00±2.00 a | 70.40±3.05 b | 46.20±3.03 a |
| | $1\times10^{-2}$ | 0.60±0.89 c | 8.40±2.30 c | ND | 17.80±2.39 c | 6.80±1.30 c | 35.80±3.42 c | 13.60±2.70 d | 48.00±3.46 c | 28.80±1.79 c |
| | $1\times10^{-1}$ | ND | 4.80±2.59 d | ND | 10.80±3.96 d | ND | 21.20±3.03 d | 2.60±1.14 e | 28.40±2.97 d | 11.40±2.41 d |
| | 1 | ND | ND | ND | 1.00±1.00 e | ND | 9.20±2.59 e | ND | 15.60±1.82 e | 6.00±1.58 e |
| | CK | 3.60±0.89 b | 22.40±1.82 a | 8.40±0.55 a | 40.20±1.64 a | 24.80±1.48 a | 60.20±1.79 a | 34.60±0.55 b | 71.20±1.92 b | 40.40±1.14 b |

**Note:** Data in the table represent mean ± standard deviation. Different lowercase letters within the same column indicate statistically significant differences ($p \leq 0.05$) in germination rates between treatments at the same germination time, while identical lowercase letters denote non-significant differences ($p > 0.05$). 'ND ' means not detected.
